# Deubiquitomic and bioinformatic analyses in cisplatin-treated lung cancer cells

**DOI:** 10.7150/ijms.120464

**Published:** 2026-01-01

**Authors:** Sun-Kyu Jin, Tae-Woo Kim, Hae-Seul Choi, Chae-Won Lee, Kwang-Hyun Baek

**Affiliations:** 1 Department of Biomedical Science, CHA University, Gyeonggi-Do 13488, Republic of Korea.; 2 Department of Bioconvergence, Graduate School, CHA University, Gyeonggi-Do 13488, Republic of Korea.; 3 Department of Biotechnology, CHA University, Gyeonggi-Do 13488, Republic of Korea.; 4 International Ubiquitin Center, Gyeonggi-Do 13488, Republic of Korea.

**Keywords:** cisplatin resistance, deubiquitinating enzymes, lung cancer biomarker, non-small cell lung cancer, ubiquitin-proteasome system

## Abstract

**Introduction**: Lung cancer is a highly lethal disease characterized by a significant mortality rate. Cisplatin, a common drug used for lung cancer treatment, frequently develops resistance over time. Therefore, overcoming cisplatin resistance is crucial in the effective management of lung cancer. The ubiquitin-proteasome system (UPS) serves as a vital regulatory mechanism for maintaining protein homeostasis within cells. Recent studies have shown that manipulating deubiquitinating enzymes (DUBs) can overcome cisplatin resistance. This study aims to investigate the expression levels of DUBs under cisplatin treatment.

**Methods**: Multiplex RT-PCR analysis was performed to identify potential biomarkers by comparing the differential expression patterns of DUBs, and their expression levels were analyzed by RT-qPCR. In addition, their protein expression levels were determined by western blot analysis. The bioinformatics tools including TCGA database and GEPIA website were used to validate potential as prognostic markers in lung cancer.

**Results**: Multiplex RT-PCR analysis was performed to identify potential biomarkers by comparing the differential expression patterns of DUB genes. Multiplex RT-PCR showed distinct mRNA expression profiles of several DUB genes, including USP35, USP36, USP37, USP47, USP49, and OTUD6B in A549 lung cancer cells following exposure to cisplatin. In addition, RT-qPCR analysis revealed the downregulation of USP35, USP36, USP37, USP47, USP49, and OTUD6B, juxtaposed with the upregulation of USP47 under cisplatin treatment. Substantiating these findings, western blotting analysis confirmed the protein expression levels of USP35, USP36, USP37, USP47, USP49, and OTUD6B in cisplatin-treated lung cancer cells, mirroring the mRNA trends observed in non-treated counterparts except for OTUD6B. Bioinformatics analysis demonstrates that these DUBs except USP47 are upregulated and overall survival analysis indicates that lower expression of these DUBs, except USP37 and USP49, is correlated with improved overall survival in lung cancer patients.

**Conclusion**: These findings strongly suggest that DUBs may play a crucial role in overcoming cisplatin resistance and improving the treatment efficacy for lung cancer.

## Introduction

The ubiquitin-proteasome system (UPS) orchestrates the intricate regulation of protein homeostasis, crucial for maintaining cellular integrity and function. This process involves the attachment of ubiquitin chains to substrates mediated by ubiquitin ligases (E3), subsequent to activation by ubiquitin-activating enzymes (E1) and transfer to ubiquitin-conjugating enzymes (E2) 1. Although many proteins tagged with ubiquitin chains undergo degradation via 26S proteasomes, not all follow this route. Ubiquitin forms various chains through seven lysine (K) residues (K6, K11, K27, K29, K33, K48, and K63) and one methionine residue (M1), with K11-, K29-, and K48-linked chains notably involved in proteasomal degradation 2, 3. Deubiquitinating enzymes (DUBs), surpassing a hundred in the human genome, counterbalance this process by removing ubiquitin from substrates 4.

As key components of the UPS, DUBs not only maintain cellular protein homeostasis but also regulate various intracellular processes, including cell proliferation, cell cycle progression, differentiation, gene expression, and signaling pathways. Consequently, they have emerged as promising therapeutic targets. With growing recognition of the importance of the UPS, increasing attention has been directed toward its role in drug resistance, oncogenesis, and cancer progression 5, 6. Numerous studies have investigated DUBs to overcome drug resistance, and attempts have been made to develop small-molecule inhibitors that inhibit DUB activity and exert synergistic effects with conventional anticancer drugs. Collectively, these studies and clinical trials strongly suggest that targeting the interplay between DUBs and drug resistance holds great potential as a novel anticancer strategy 3.

Cancer stands as a foremost global cause of morality, with lung cancer representing a particular challenging entity characterized by poor prognoses, boasting one of the lowest 5-year survival rates 7. Lung cancer is categorized into small cell lung cancer (SCLC) and non-small cell lung cancer (NSCLC), with the latter comprising the majority of cases (80-85%). NSCLC further divides into adenocarcinoma (ADC; ~40%), squamous cell carcinoma (SCC; 25-30%), and large cell carcinoma (LCC; 10-15%) 8-11.

Platinum-based chemotherapy, particularly cisplatin, plays a pivotal role in cancer treatment. These drugs exert their effects by binding to guanine bases of DNA, thereby inhibiting DNA synthesis and ultimately inducing cell death 12-14. Cisplatin, the first-generation platinum compound, serves as a cornerstone in the treatment of various tumors 15. Its cellular uptake is mediated by membrane proteins such as multidrug resistance protein (MRP), ATPase copper transporting alpha/beta (ATP7A/B), and high affinity copper uptake protein 1 (CTR1). Once inside the cell, cisplatin undergoes hydroxylation, followed by covalent bonding to DNA bases, especially at the N7 position of guanine. This process disrupts transcription and DNA synthesis, leading to cell cycle arrest and triggering DNA repair mechanisms or apoptosis 16. However, prolonged administration of anticancer drugs often causes resistance, posing a formidable challenge in cancer management 17. Mechanisms underlying this resistance include increased expression of proteins that facilitate drug efflux, enhanced DNA damage repair, and inactivation of the drug by molecules such as glutathione (GSH), metallothionein, excision repair cross complementing protein 1 (ERCC1), xeroderma pigmentosum complementation group F (XPF), and Breast Cancer gene 1 and Breast Cancer gene 2 (BRCA1/BRCA2) 18, 19. These mechanisms interact in complex ways, making it difficult to define a single dominant cause of cisplatin resistance.

In a notable study, cisplatin exhibited effectiveness in about 20-40% of NSCLC patients when administered in combination with other anticancer drugs 13. However, recurrence within six months was observed in the majority of patients, indicative of the emergence of cisplatin resistance during chemotherapy.

In this study, we screened differentially expressed *DUB* genes following cisplatin treatment to identify potential genes involved in either inducing or decreasing cisplatin resistance. Multiplex RT-PCR revealed distinct expression patterns of *USP35*,* USP36*,* USP37*, *USP47*, *USP49,* and *OTUD6B* compared to non-treated cells. These DUBs are likely to participate in processes such as DNA repair, apoptosis, and cisplatin resistance, thereby shedding light on potential therapeutic targets.

## Materials and Methods

### Cell culture and cisplatin treatment

A549 cells (CCL-185, ATCC, Manassas, VA, USA) were cultured in DMEM (12800-017, Gibco, Grand Island, NY, USA) and H1299 cells (CRL-5803, ATCC, Manassas, VA, USA) were grown RPMI-1640 medium (11875-093, Gibco BRL, Rockville, MD, USA) adding 10% fetal bovine serum (FBS, 12483020, Gibco, Grand Island, NY, USA) and 1% penicillin/streptomycin (PS, 15140122, Gibco, Grand Island, NY, USA). The cells were cultured at 37°C in a humidified atmosphere containing 5% CO_2_. To investigate the expression levels of *DUB* genes under cisplatin (232120, Sigma-Aldrich, St. Louis, MO, USA) treatment, For multiplex RT-PCR and RP-qPCR, 60 µM of cisplatin was treated for 24 hrs. Additionally, for western blotting analysis, A549 and H1299 cells were treated with varying concentration of cisplatin (0, 15, 30, and 45 µM) for 24 hrs.

### RNA extraction, cDNA synthesis, multiplex RT-PCR, and RT-qPCR

For RNA extraction, the cells were lysed by TRIzol reagent (15596018, Thermo Fisher Scientific, Waltham, MA, USA) in a culture dish. cDNA synthesis was carried out using the LaboPass™ cDNA synthesis kit (CMRTK001, Cosmogenetech, Seoul, Korea) following the manufacturer's protocol. *GAPDH* was employed as an internal standard. For multiplex RT-PCR, Multiplex PCR pre-mix (SMP01-M25h, SolGent, Daejeon, Korea) was used, and cDNAs were amplified according to the manufacturer's protocol. *GAPDH* was used as a control. The PCR products were loaded into 2.5% agarose gel stained with RedSafe DNA Stain (21141, Chembio, Medford, NY, USA) for electrophoresis. Primer sequences for multiplex PCR were obtained from prior literature 20. Quantitative PCR was performed with SYBR Green PCR Master Mix (4309155, Applied Biosystems, Thermo Fisher Scientific, MA, USA) using the synthesized cDNAs (The RT-qPCR primers are listed in Supplementary Table 1). The expression of each gene was normalized to *GAPDH*, and relative changes were calculated by 2^-ΔΔCt^ method.

### Western blot analysis and antibodies

Cells were lysed with lysis buffer (50 mM Tris-HCl [pH 7.5], 1 mM EDTA, 300 mM NaCl, 10% glycerol, and 1% Triton X-100). The samples were ice-incubated for 20 min and then centrifuged for 20 min at 4°C (13,000 rpm). The supernatants containing protein extracts were collected and boiled with 2X SDS buffer. Samples were loaded onto sodium dodecyl-sulfate polyacrylamide gel electrophoresis (SDS-PAGE) gels. Proteins of gels transferred onto polyvinylidene fluoride (PVDF) membranes (IPVH00010, Millipore, Billerica, MA, USA). The membranes were blocked with 5% bovine serum albumin (BSA) or 5% skim milk in TBS-T (20 mM Tris-HCl [pH 7.5], 0.05% Tween 20 and 150 mM NaCl) at room temperature for 30 min. The membranes were incubated with primary antibodies at 4°C overnight. Following primary antibody reaction, the blots were washed three times with TTBS for 7 min each and then reacted with the appropriate secondary antibody at room temperature for 1 hr. After three additional washes with TTBS, proteins were visualized using an ECL solution (LF-QC0101, Young-In Frontier, Seoul, Korea). The following primary antibodies were used: anti-β-actin (sc-4778, Santa Cruz Biotechnology, Santa Cruz, CA, USA), anti-Bax (sc-20067, Santa Cruz Biotechnology, Santa Cruz, CA, USA), anti-USP36 (68165-1-Ig, Proteintech, Rosemont, IL, USA), anti-USP37 (A15593, ABclonal, Woburn, MA, USA), anti-USP47 (sc-100633, Santa Cruz Biotechnology, Santa Cruz, CA, USA), anti-USP49 (sc-82411, Santa Cruz Biotechnology, Santa Cruz, CA, USA), and anti-OTUD6B (Q8N6M0, CUSABIO, Houston, TX, USA).

### Bioinformatics analysis

Gene Expression Profiling Interactive Analysis (GEPIA, http://gepia2.cancer-pku.cn/) and the University of Alabama at Birmingham Cancer data analysis portal (UALCAN, https://ualcan.path.uab.edu/), utilizing data from The Cancer Genome Atlas (TCGA), were used to analyze the expression levels of *DUBs* and generate survival curves, specifically overall survival (OS) in lung adenocarcinoma (LUAD) and lung squamous cell carcinoma (LUSC). Survival analysis was performed by categorizing patients into high-expression and low-expression groups, representing the top and bottom 33% based on *DUB* gene expression levels. Furthermore, a protein-protein interaction network was constructed to visualize the interactions between DUBs and their putative substrates using several bioinformatics tools, including the Biological General Repository for Interaction Datasets (BioGRID, https://thebiogrid.org), the Database for Annotation, Visualization, and Integrated Discovery (DAVID, https://davidbioinformatics.nih.gov/summary.jsp), STRING (https://string-db.org/), and Cytoscape. Protein interaction datasets for each DUB were obtained from the BioGRID repository and then converted into ENSEMBL gene ID for DAVID analysis, except for unknown genes. The gene sets identified through DAVID were then used to generate interaction networks with STRING, and the resulting maps were visualized using Cytoscape.

### Statistical analysis

Results are shown as mean ± standard deviation (SD). For statistical analysis, either an unpaired two-tailed Student's *t*-test or one-way ANOVA followed by Tukey's *post hoc* correction was applied, as appropriate (GraphPad Prism 9.0) (GraphPad Software, La Jolla, CA, USA). Band intensity was quantified through densitometric analysis using ImageJ software (https://imagej.net/ij/download.html, version 1.4.3.6). All data was obtained from at least three independent experiments. *p* values of *p*<0.05 (*), *p*<0.01 (**), or *p*<0.001 (***) were considered statistically significant.

## Results

### Multiplex RT-PCR screening of *DUB* genes in cisplatin-treated NSCLC

**Figure 1:**
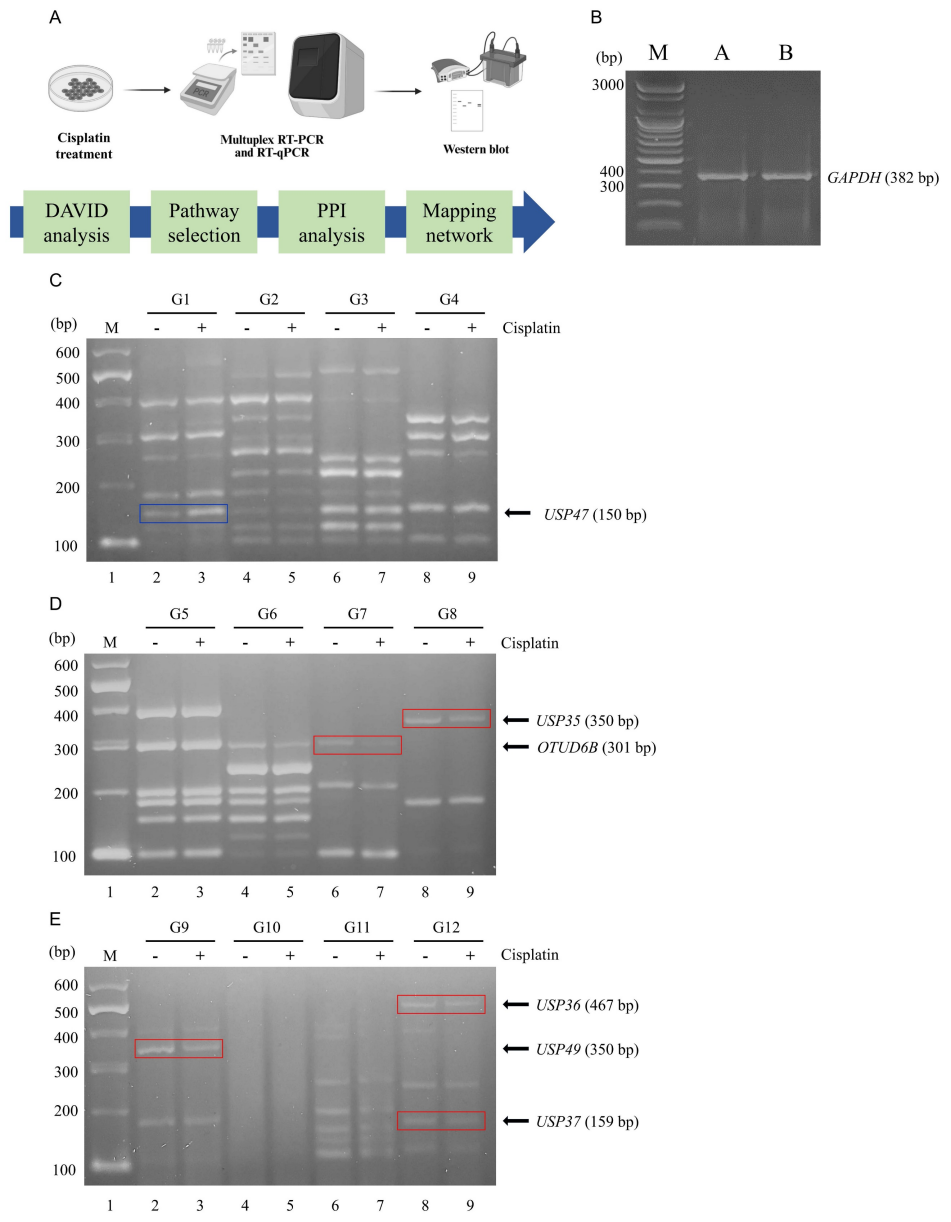
Multiplex RT-PCR screening of *DUB* genes associated with cisplatin treatment. (A) Schematic overview of the experimental workflow. Western blot and PCR machinery illustration are created with BioRender.com. (B) mRNA expression levels of *GAPDH* were determined by PCR and used as controls to normalize *DUB* expression levels obtained from multiplex RT-PCR. (C-E) Results of *DUB* screening using multiplex RT-PCR with primer sets G1 through G12. The blue line signifies an increase in *DUB* expression, while the red line indicates a decrease. G, group; M, DNA marker.

To investigate the DNA damage induced by cisplatin in lung cancer cells, A549 cells were treated with 60 μM of cisplatin. Subsequently, RNA was extracted from both untreated A549 cells and cisplatin-treated A549 cells, followed by cDNA synthesis for multiplex RT-PCR using the *DUB* gene-specific primer groups G1-G12. The overall experimental workflow is illustrated in Figure 1A. *GAPDH* expression level was determined in both cells and served as an internal control to normalizing gene expression data (Fig. 1B).

Multiplex RT-PCR products were subjected to agarose gel electrophoresis and densitometric analysis to evaluate differential expression patterns (Fig. 1C-E). The analysis revealed significant variations in the expression levels of several *DUB* genes between untreated and cisplatin-treated A549 cells. Notably, *USP47* (G1), *OTUD6B* (G7), *USP35* (G8), *USP49* (G9), *USP36* (G12), and *USP37* (G12) exhibited the most prominent differences. Specifically, the expression levels of *OTUD6B* (G7),* USP35* (G8), *USP49* (G9), *USP36* (G12), and *USP37* (G12) were downregulated in cisplatin-treated A549 cells, whereas the expression level of *USP47* (G1) was upregulated.

### Differential expression of *DUB* genes in mRNA levels upon cisplatin-treatment

**Figure 2:**
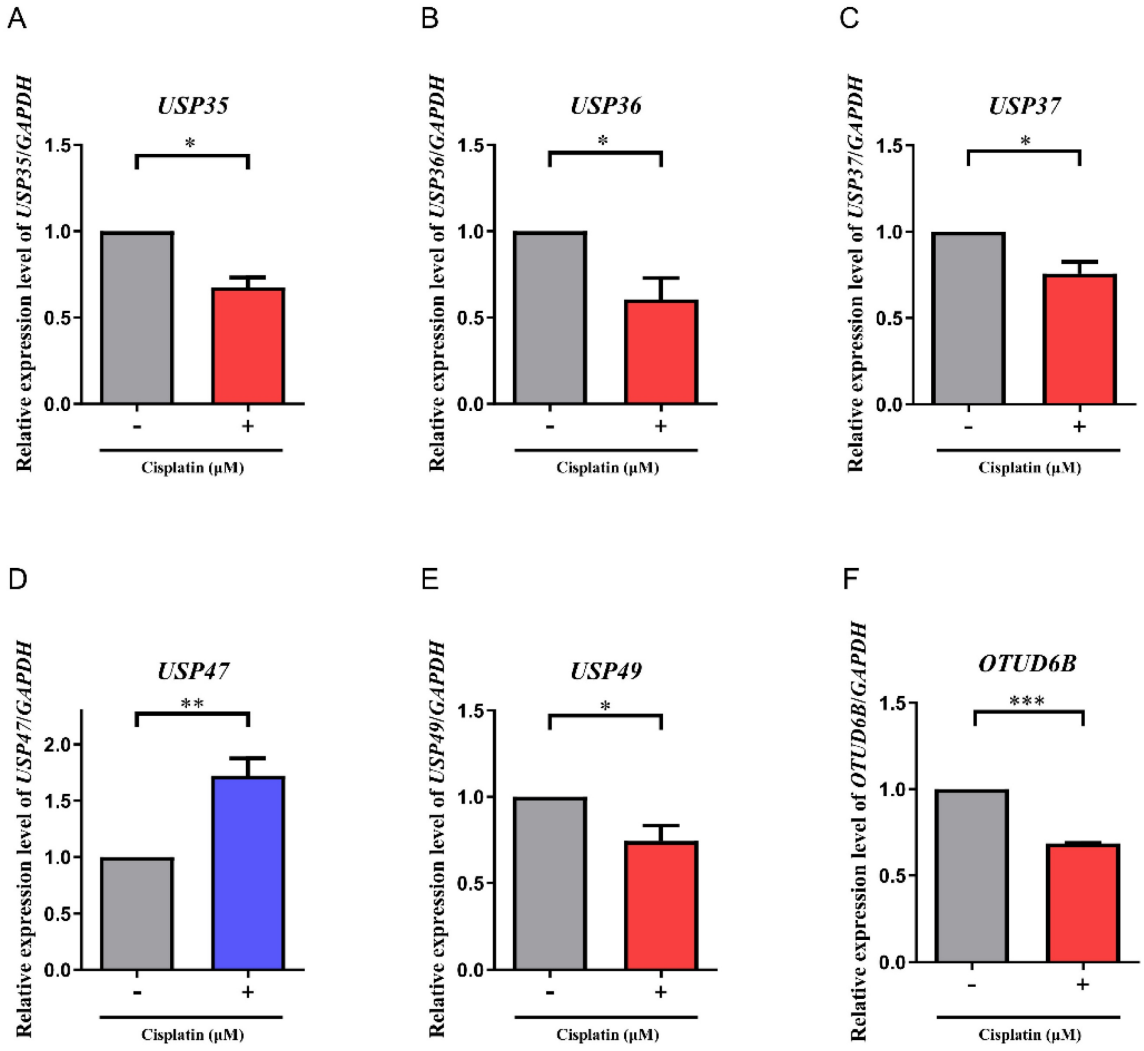
mRNA expression levels of *DUB* genes. (A-F) The mRNA expression levels of *USP35*, *USP36*, *USP37*, *USP47*, *USP49*, and *OTUD6B* were investigated by RT-qPCR. Data are presented as the mean ± standard error of the mean; n=6; *p<0.05, **p<0.01, and ***p<0.001.

To verify the findings obtained from the multiplex RT-PCR analysis, the expression levels of *USP35*, *USP36*, *USP37*,* USP47*,* USP49,* and *OTUD6B* were further investigated using RT-qPCR (Fig. 2A-F). Consistent with the results of the multiplex RT-PCR, RT-qPCR analysis revealed similar trends in gene expression. Specifically, the expression levels of* USP35*, *USP36*, *USP37*,* USP49*, and *OTUD6B* in cisplatin-treated A549 cells were reduced by approximately 0.64-fold, 0.56-fold, 0.72-fold, 0.66-fold, and 0.68-fold, respectively, compared to non-treated A549 cells. Conversely, the expression level of *USP47* was elevated by approximately 1.72-fold in cisplatin-treated A549 cells compared to non-treated counterparts.

### Differential expression of DUBs in protein levels upon cisplatin-treatment

**Figure 3:**
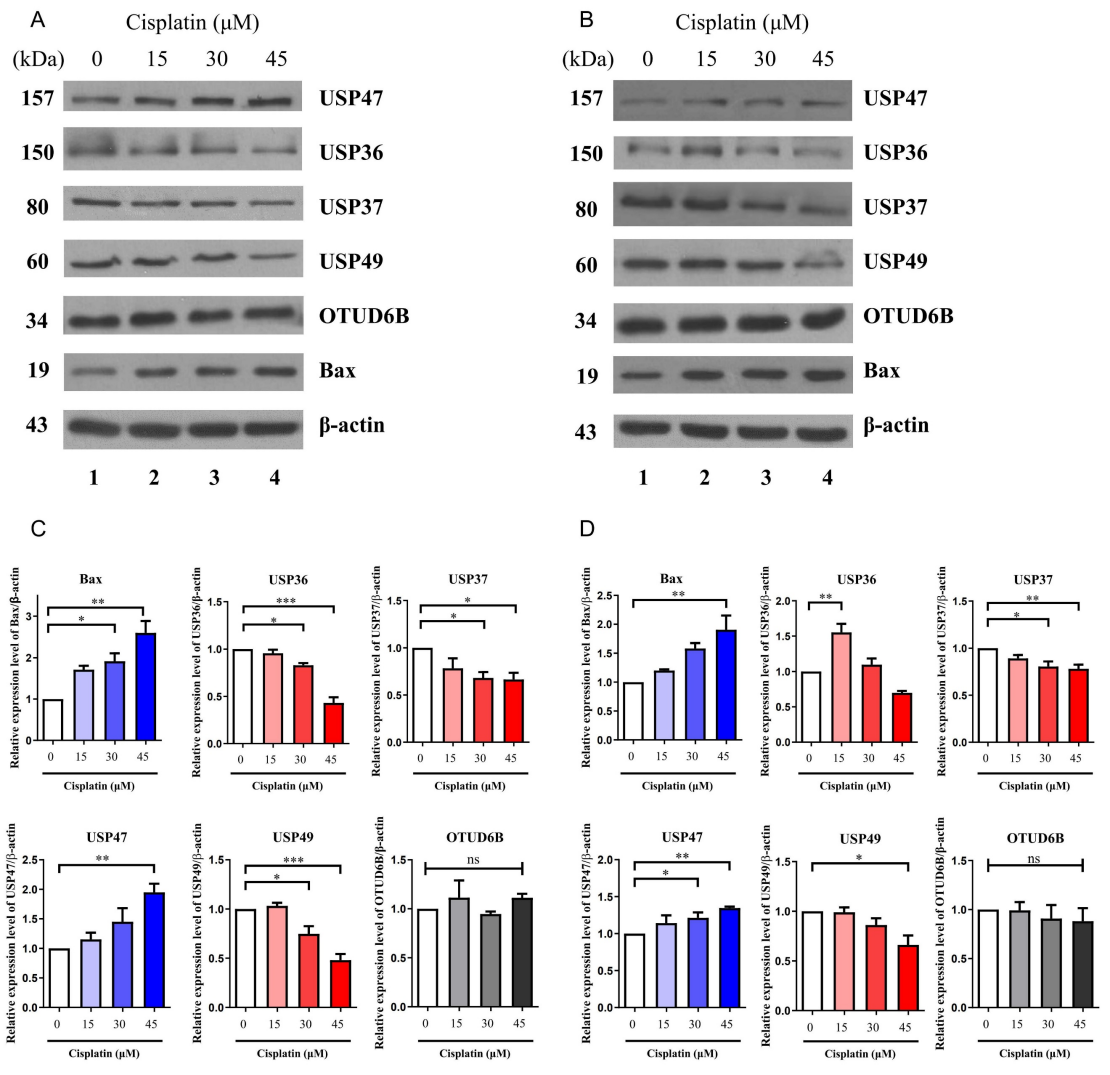
Protein expression levels of DUBs. (A) Western blot analysis of USP36, USP37, USP47, USP49, and OTUD6B in A549 cells. Experiments were conducted at least three times. (B) Western blot analysis of USP36, USP37, USP47, USP49, and OTUD6B in H1299 cells. Experiments were conducted at least three times. (C, D) Quantification of USP36, USP37, USP47, USP49, and OTUD6B protein levels using ImageJ and GraphPad Prism 9.0. Data are presented as the mean ± standard error of the mean; n=4; *p<0.05, **p<0.01, ***p<0.001, and ns; not significant.

While mRNA levels can provide insights into protein expression, but there is often no direct correlation between the two. Additionally, DUBs play a critical role in regulating protein stability through post-translational modifications. A recent study reported that suppression of USP35 makes lung cancer cells sensitive to cisplatin 21. Therefore, the protein expression levels of USP36, USP37, USP47, USP49, and OTUD6B were examined via western blotting in A549 and H1299 cells treated with increasing concentrations of cisplatin. To confirm cisplatin-induced apoptosis, the expression level of the apoptotic protein, BCL2-associated X (Bax), was also assessed in a dose-dependent manner. The results revealed a decrease in the expression of USP36, USP37, and USP49 with increasing concentration of cisplatin in both cell lines, while the expression of USP47 increased under the same conditions (Fig. 3A-D). However, no significant change in the expression of OTUD6B was observed. These findings were consistent with the trends observed in mRNA expression levels, except for OTUD6B.

### Association of *DUB* expression levels with overall survival rate

**Figure 4:**
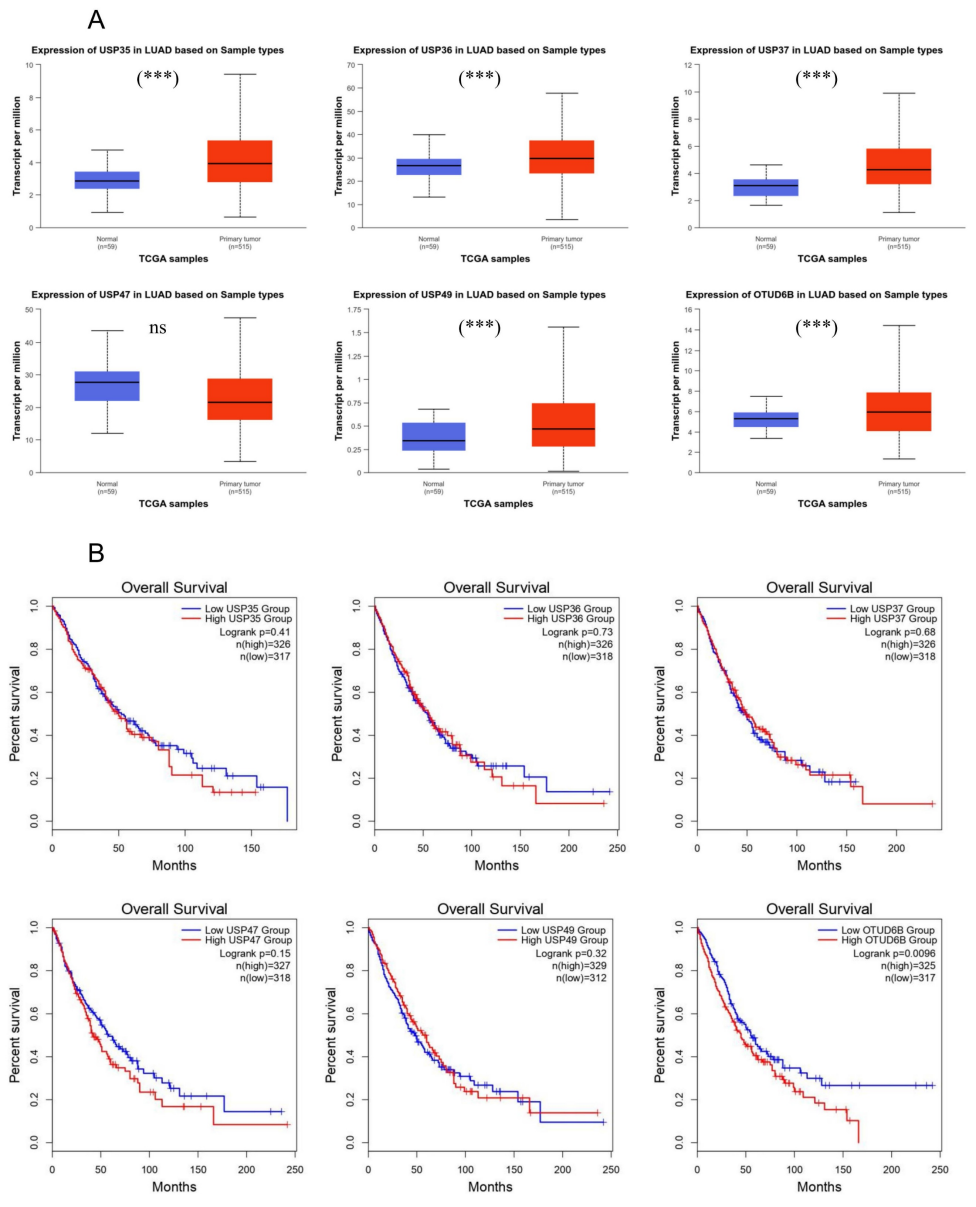
Association of *DUB* expression levels with overall survival. (A) Analysis of *USP35*, *USP36*, *USP37*, *USP47*, *USP49*, and *OTUD6B* expression in LUAD using the UALCAN platform based on TCGA database. (B) Effects of *USP35*, *USP36*, *USP37*, *USP47*, *USP49*, and *OTUD6B* expression on overall survival in LUAD and LUSC, analyzed using the GEPIA database. Data are presented as the mean ± standard error of the mean; ***p<0.001 and ns; not significant.

Following the observation of altered expression levels of USP35, USP36, USP37, USP47, USP49, and OTUD6B in response to cisplatin treatment in lung cancer cells, both at the mRNA and protein levels, we sought to explore their significance in lung cancer. Using the GEPIA website, which draws data from TCGA, we compared the expression levels of these DUBs between normal and lung cancer patients (Fig. 4A). Our analysis revealed an upregulation of *USP35*, *USP36*, *USP37*, *USP49*, and *OTUD6B*, whereas *USP47* exhibited downregulation in the patient group. To further investigate the prognostic potential of these DUBs in lung cancer, we assessed their prognostic values, also based on TCGA data (Fig. 4B). With the exception of *USP37* and *USP49*, lower expression levels of the identified DUBs were associated with increased survival rates. These findings suggest a potential role for USP35, USP36, USP47, and OTUD6B as prognostic markers in lung cancer.

### Analysis of putative substrates using bioinformatics tools

**Figure 5:**
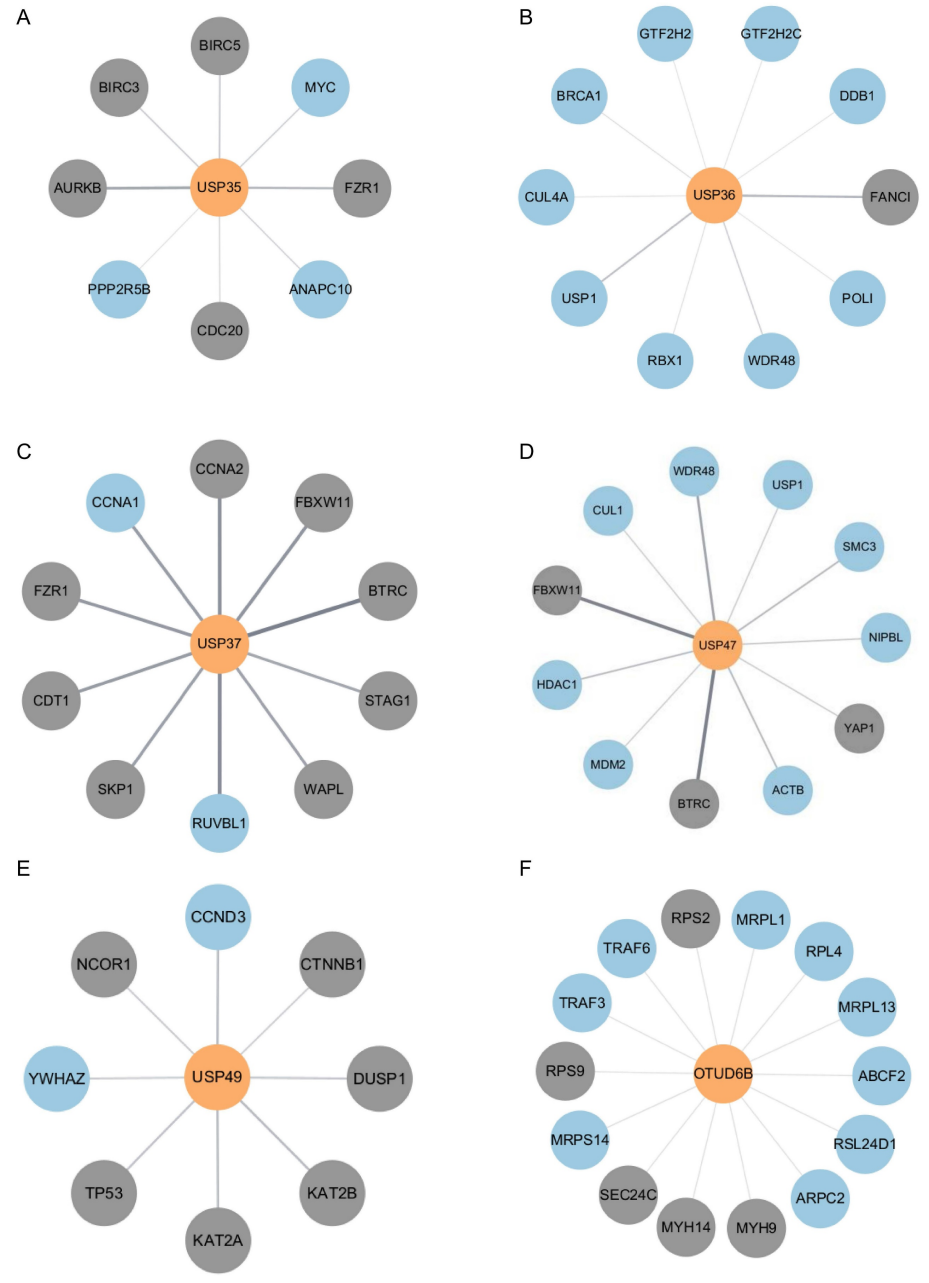
Network maps illustrating interactions between each DUB and its pustative substrates. (A) Interaction network between USP35 and putative substrates. (B) Interaction network between USP36 and putative substrates. (C) Interaction network between USP37 and putative substrates. (D) Interaction network between USP47 and putative substrates. (E) Interaction network between USP49 and putative substrates. (F) Interaction network between OTUD6B and putative substrates. The color of nodes indicates the confirmation of binding between DUB and proteins (Gray: identified and blue: non-identified).

Given that the DUBs identified in the above experiments may regulate substrates involved in the mechanism of cisplatin action, we investigated putative substrates for USP35, USP36, USP37, USP47, USP49, and OTUD6B via bioinformatics-based protein-protein interaction (PPI) analysis. Using gene datasets obtained from DAVID analysis, we constructed interaction networks between each DUB and its putative substrates using the STRING database, and visualized these networks with Cytoscape software (Fig. 5A-F). Our analysis focused specifically on pathways related to cell proliferation and DNA damage repair, which are central to the mechanism of cisplatin action. As a result, previously reported substrates of each DUB were excluded. These analyses provide insights into the molecular pathways by which DUBs may modulate cisplatin responses.

## Discussion

In our study, we identified expression profile of DUB both mRNA and protein level, revealing notable changes in the expression levels of USP35, USP36, USP37, USP47, USP49, and OTUD6B after cisplatin treatment. Numerous studies have delved into the intricate relationship between DUBs and various cancers, including NSCLC. DUBs, owing to their diverse substrate regulatory roles across numerous signaling pathways, have garnered considerable attention in cancer research 22, 23. Efforts to develop DUB inhibitors have progressed to clinical trials, underscoring their potential as therapeutic targets 24, 25. Therefore, elucidating the functions and mechanisms of specific DUBs in NSCLC holds promise for identifying novel strategies to overcome the resistance of anticancer drugs.

USP35, USP36, USP37, USP47, USP49, and OTUD6B play crucial roles in cancer progression and chemoresistance across various cancers, including NSCLC. These DUBs modulate numerous cellular processes, including DNA replication stress responses, endoplasmic reticulum (ER) stress, epithelial-mesenchymal transition (EMT), phosphatidylinositol 3-kinase (PI3K)/protein kinase B (AKT) pathway, and metabolic processes 22. Among these DUBs, USP35 regulates cisplatin resistance through deubiquitination of baculoviral IAP repeat containing 3 (BIRC3) and ferroportin (FPN) in NSCLC. Knockdown of USP35 has been shown to sensitize lung cancer cells to cisplatin by enhancing apoptosis and ferroptosis via BIRC3 and FRN, respectively 21, 26. USP36, USP37, USP47, and OTUD6B exert its oncogenic effects by stabilizing key oncoproteins, such as cellular myelocytomatosis oncogene (c-Myc), 14-3-3γ, Snail, and β-catenin, thereby promoting cancer cell proliferation, migration, and invasion 27-32. Besides, USP35, USP36, and USP47 have been shown to promote cancer cell proliferation and development by interacting and deubiquitinating ribosome-binding protein 1 (RRBP1), primase and DNA directed polymerase (PrimPol), and BTB and CNC homology 1 (BACH1), mitigating ER stress-induced apoptosis and DNA replication stress caused by cisplatin and promoting Warburg effect, respectively 33-35. On the other hand, USP49, known for its tumor-suppressive role, has been shown to suppress the activation of PI3K/AKT signaling by deubiquitinating phosphatase and tensin homolog (PTEN) and FK506-binding protein 51 (FKBP51), thereby inhibiting cancer cell proliferation 36, 37. Given that DUBs can modulate the activity of membrane proteins and proteins involved in DNA repair processes, it is plausible that the DUBs identified in our study may have substrates implicated in the cisplatin mechanism. Investigating these potential substrates could offer valuable insights into the molecular mechanisms underlying cisplatin resistance in lung cancer and may reveal novel therapeutic targets for intervention.

In our study, we observed significant changes in the expression levels of USP35, USP36, USP37, USP47, USP49, and OTUD6B following cisplatin treatment, which were consistent at the protein level, except for OTUD6B. While *OTUD6B* exhibited decreased mRNA expression after cisplatin treatment, there was no corresponding change in protein expression. This discrepancy between mRNA and protein levels highlights the complexity of gene regulation and protein synthesis processes. It is noteworthy that while mRNA and protein expression levels often correlate, various factors such as transcript length, cellular state, and translational efficiency can influence this relationship. Moreover, translation is a highly regulated process that requires energy, and under conditions of stress like cisplatin treatment, translational activity may be modulated to meet cellular demands. Consequently, transcripts may not be uniformly translated into proteins, leading to disparities between mRNA and protein levels 38, 39.

In addition, we evaluated the prognostic significance of the identified DUBs in lung cancer patients using the TCGA database. Interestingly, with the exception of USP37 and USP49, lower expression levels of most candidate DUBs were associated with improved overall survival. However, the survival analysis of these DUBs except OTUD6B did not reach statistical significance. These findings suggest that DUB expressions may serve as a potential diagnostic marker for predicting prognosis in lung cancer patients. Further validation using *in vivo* or patient samples will be essential to establish their clinical utility as prognostic indicators.

In the current study, we also constructed protein-protein interaction networks between DUBs and their putative substrates using bioinformatics tools. Since bioinformatics can be used as an indispensable tool in analyzing not only proteins but also chemical and genomic interactomes across large datasets, our results highlight its value in bridging molecular biology and computational approaches. This suggests that the potential of bioinformatics provides novel insights into molecular mechanisms and significantly contributes to the study of protein interactomes.

## Conclusion

In this study, our findings shed light on the complex interplay between DUB expression, cisplatin treatment, and lung cancer biology. Future studies aimed at unraveling the mechanistic roles of DUBs in cisplatin response are warranted and may pave the way for the development of targeted therapies to mitigate cisplatin resistance in lung cancer.

## Supplementary Material

Supplementary figures and tables.

## Abbreviations

UPS: ubiquitin-proteasome system
DUBs: Deubiquitinating enzymes
SCLC: small cell lung cancer
NSCLC: non-small cell lung cancer
ADC: adenocarcinoma
SCC: squamous cell carcinoma
LCC: large cell carcinoma
MRP: multidrug resistance protein
ATP7A/B: ATPase copper transporting alpha/beta
CTR1: copper uptake protein 1
GSH: glutathione
ERCC1: excision repair cross complementing protein 1
XPF: xeroderma pigmentosum complementation group F
BRCA1/2: Breast Cancer gene 1 and Breast Cancer gene 2
ER: endoplasmic reticulum
EMT: epithelial-mesenchymal transition
PI3K: phosphatidylinositol 3-kinase
AKT: protein kinase B
BIRC3: baculoviral IAP repeat containing 3
FPN: ferroportin
c-Myc: cellular myelocytomatosis oncogene
RRBP1: ribosome-binding protein 1
PrimPol: primase and DNA directed polymerase
BACH1: BTB and CNC homology 1
PTEN: phosphatase and tensin homolog
FKBP51: FK506-binding protein 51
GEPIA: Gene Expression Profiling Interactive Analysis
UALCAN: University of Alabama at Birmingham Cancer data analysis portal
OS: overall survival
LUAD: lung adenocarcinoma
LUSC: lung squamous cell carcinoma
TCGA: The Cancer Genome Atlas
PPI: protein-protein interaction
BioGRID: Biological General Repository for Interaction Datasets
DAVID: database for annotation, visualization, and integrated discovery
