# Supplementary material for: Deubiquitomic and bioinformatic analyses in cisplatin-treated lung cancer cells
Source: Int J Med Sci. 2026 Jan 1;23(1):1–11. doi: 10.7150/ijms.120464 (PMC12702012; doi:10.7150/ijms.120464)
Supplement: Supplementary file 1 — Supplementary figures and tables. [file ijmsv23p0001s1.pdf]

Table 1. Primers used for RT-qPCR.

DUB gene		Primer Sequence (5′→3′)
USP35	FP:	AAG TAC ATG CTC CTG ACC TTC CA
	RP:	CCC AGG TTG ATG AGA CCA ATC TT
USP36	FP:	TCC CAG ACA CCC ACA CAC AT
	RP:	GTG GTG TTG CTC AGG ACA GG
USP37	FP:	CAG AAG GAA ACC AGC AGG CA
	RP:	CGT CCG AGC TAT TCC ACT TCC
USP47	FP:	CGA TGA TCA ACA TGT CAG CAG GA
	RP:	TTT CTG GCT GGA TCC TTC AGT CT
USP49	FP:	AGG ACT ACG TGC TCA ATG ATA ACC
	RP:	GCA GGA GCA GCC GTG CAC TCT
OTUD6B	FP:	AAG AAT GCT GTT CCC AAG AA
	RP:	CCA TAT GTC TGG CTC CTG TT
GAPDH	FP:	CCC TGA ACG CTA TCA CTG CA
	RP:	CCA TCA CGC CAC AGT TTC
